# Cold‐Induced Suppression of Myogenesis in Skeletal Muscle Stem Cells Contributes to Delayed Muscle Regeneration During Hibernation

**DOI:** 10.1096/fj.202502651R

**Published:** 2025-12-01

**Authors:** Tatsuya Miyaji, Ryuichi Kasuya, Mayuko Monden, Yutaka Tamura, Michito Shimozuru, Toshio Tsubota, Daisuke Tsukamoto, Guangyuan Li, Shota Kawano, Yuri Watanabe, Yoshifumi Yamaguchi, Masatomo Watanabe, Mitsunori Miyazaki

**Affiliations:** ^1^ Department of Integrative Physiology, Graduate School of Biomedical and Health Sciences Hiroshima University Hiroshima Japan; ^2^ Department of Pharmacology, Faculty of Pharmacy and Pharmaceutical Sciences Fukuyama University Fukuyama Japan; ^3^ Laboratory of Wildlife Biology and Medicine, Faculty of Veterinary Medicine Hokkaido University Sapporo Japan; ^4^ Laboratory of Molecular Biology, Department of Biosciences, School of Science Kitasato University Sagamihara Kanagawa Japan; ^5^ Hibernation Metabolism, Physiology, and Development Group, Environmental Biology Division Institute of Low Temperature Science, Hokkaido University Sapporo Japan

**Keywords:** cold stress, ferroptosis, hibernation, muscle regeneration, myogenesis, satellite cells, skeletal muscle

## Abstract

Mammalian hibernators experience profound cold stress and prolonged physical inactivity during torpor periods; however, it is unclear how skeletal muscle stem cells (satellite cells; SCs) respond to these challenges. In this study, we demonstrated that SCs from a mammalian hibernator, the Syrian hamster, exhibit remarkable resistance to cold‐induced cell death, which is associated with intrinsically higher expression of the antioxidant enzyme GPX4, likely contributing to ferroptosis suppression. RNA‐seq analysis revealed widespread downregulation of myogenesis‐related genes following cold exposure, suggesting suppression of the myogenic program. Consistently, SCs exposed to cold stress exhibited reduced activation and differentiation capacity upon subsequent rewarming, with an increased number of quiescent Pax7‐positive/MyoD‐negative cells. Muscle regeneration was markedly delayed during hibernation, accompanied by decreased SC activation and macrophage infiltration, suggesting that cold‐induced suppression of SC function underlies the limited regenerative capacity in hibernating hamsters. Our results provide insights into the unique physiology of mammalian hibernators: SC viability is preserved, whereas regenerative activity is selectively suppressed during hibernation.

## Graphical abstract

Hibernating mammals preserve satellite cell viability during extreme cold exposure by suppressing ferroptosis through elevated GPX4 expression. Although these cells survive cold stress, myogenic activation and differentiation are markedly reduced, leading to delayed muscle regeneration in vivo. Cold exposure shifts satellite cells toward a quiescent Pax7^+^/MyoD^−^ state, while regenerative and inflammatory responses are attenuated during hibernation. These findings highlight a dual strategy in which stem cell integrity is maintained but energy‐demanding regenerative programs are selectively downregulated.

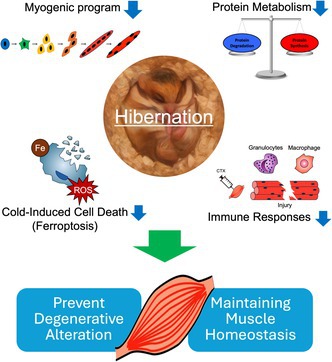

## 1 Introduction

Hibernation is a survival strategy that organisms have developed throughout evolution to survive the harsh winter environment of cold ambient temperature (*T*_a_) and food deprivation. “Torpor” refers to the active reduction of energy metabolism and body temperature (*T*_b_) under conditions of cold and energy depletion in homeotherms that essentially maintain a constant *T*_b_ [1, 2, 3]. Prolonged periods of torpor lasting for multiple days, occurring in a seasonal manner, are generally defined as hibernation [1, 2, 4, 5]. There are more than 180 hibernating mammalian species belonging to seven orders (*Monotremata*, *Marsupialia*, *Insectivora*, *Chiroptera*, *Primates*, *Rodentia*, and *Carnivora*), which indicates that approximately 5%–6% of all mammals use this survival strategy [1, 2, 5]. In large hibernators such as bears, *T*_b_ decreases to approximately 30°C–36°C during hibernation, and the metabolic rate is suppressed to approximately 25% of the basal metabolic rate (BMR) [6]. In small hibernators such as hamsters and chipmunks, *T*_b_ decreases to near *T*_a_, often as low as 2°C–5°C during torpor [7, 8]. Heart rate, respiratory rate, and whole‐body energy metabolism are markedly reduced (2%–5% of BMR) [9, 10]. These animals also experience rewarming of T_b_ during periodic arousal, thus repeating the torpor‐arousal cycle every few days during hibernation [11, 12, 13]. Despite exposure to prolonged periods of cold stress, hibernating animals maintain physiological homeostasis without developing organ dysfunction [14].

Living organisms are exposed to a variety of environmental stresses, including temperature changes. Excessive cellular stress can trigger cell death leading to tissue damage and organ dysfunction. Cold‐induced cell death (CICD), induced by prolonged exposure to hypothermic conditions, is mediated by ferroptosis [15]. Ferroptosis is a regulated form of cell death characterized by lipid peroxidation and iron dependency [16]. Syrian hamster‐derived cultured cell lines exhibit resistance to CICD through the regulation of glutathione peroxidase 4 (GPX4), a glutathione‐dependent antioxidant enzyme and a key regulator of ferroptosis [17, 18]. In addition, primary hepatocytes from Syrian hamsters are resistant to CICD [19]. Although such resistance may enable Syrian hamsters to survive extremely cold conditions during hibernation, it remains unclear whether other tissues or hibernating species contain comparable mechanisms through ferroptosis suppression. Furthermore, the adaptive responses exhibited by surviving cells in hibernating animals following cold exposure are poorly understood.

One remarkable feature of mammalian hibernators is their ability to preserve skeletal muscle mass despite experiencing prolonged physical inactivity [20]. Skeletal muscle is the largest organ in the body and plays an important role in force production and energy metabolism. Muscle loss due to aging or disuse has serious consequences for human health [21, 22]. Satellite cells (SCs), the resident stem cells of skeletal muscle, are essential for muscle maintenance, repair, and regeneration [23, 24, 25]; however, it is unclear how SCs in mammalian hibernators adapt to cold exposure. Although the cellular bases that allow skeletal muscle to tolerate prolonged cold exposure in hibernators have not been elucidated, we considered that investigating how SCs respond to extreme cold stress would provide important mechanistic insight into muscle preservation during hibernation. Therefore, in this study, we isolated SCs from multiple mammalian hibernators and examined their responses to extreme cold exposure both in vitro and in vivo. We hypothesized that SCs from hibernating species possess intrinsic mechanisms that allow them to survive extreme cold stress by suppressing CICD and maintaining their regenerative potential upon rewarming. Understanding these adaptive mechanisms may also provide broader insight into how low‐temperature environments affect muscle maintenance, and may ultimately inform strategies for protecting muscle under conditions such as aging, disuse, or therapeutic hypothermia.

## 2 Materials and Methods

All reagents, antibodies, and commercially available materials used in this study are listed in Table S1.

### 2.1 Animals

All animal procedures were approved by the Animal Ethics and Research Committee of Hiroshima University (No. A22‐7‐6 and No. A24‐38‐2), Fukuyama University (No. 2022‐A‐12 and 2025‐A‐5), Hokkaido University (No. 23‐0144), and Kitasato University (No. SA2402) and complied with the institutional guidelines. The hamsters (Slc:Syrian), mice (C57BL/6JmsSlc), and rats (Slc:SD) used for SC isolation were sexually mature (8–16 weeks old) males. They were purchased from Japan SLC (Shizuoka, Japan) and maintained in a temperature‐ and humidity‐controlled animal facility (23°C ± 1°C, 50%–60% humidity) at Hiroshima University under a 12 h light/12 h dark cycle. They were provided autoclaved water and a certified rodent diet ad libitum. Skeletal muscle samples were harvested under anesthesia, followed by euthanasia (mice: cervical dislocation under 2.0% isoflurane; rats and hamsters: intraperitoneal overdose of sodium pentobarbital at 200 mg/kg).

For the hibernation experiments, Syrian hamsters were bred at Fukuyama University using breeding pairs (Slc:Syrian) obtained from Japan SLC (Shizuoka, Japan). All animals were male and housed under standard conditions (23°C ± 1°C, 14 h light/10 h dark cycle) until 8 weeks of age, at which time they were intraperitoneally implanted with a temperature logger. After a 1‐week recovery period, they were transferred to a controlled environment (5°C, 8 h light/16 h dark) designed to simulate a winter‐like environment to induce hibernation. The animals were individually housed with *ad libitum* access to diets (Labo MR Standard, Nihon Nosan, Japan). The duration required for entry into hibernation varied among individuals.

The chipmunks ( *Tamias sibiricus* ) used for skeletal muscle collection were approximately 6 months old males, purchased from Pure Animal (Tokyo, Japan). They were individually housed at 23°C under a 12 h light/12 h dark cycle in the animal facility at Kitasato University, with free access to a standard rodent diet and water. Skeletal muscle samples were collected from non‐hibernating individuals euthanized under deep isoflurane anesthesia.

Skeletal muscle samples from Asiatic black bears ( *Ursus thibetanus japonicus* ) were obtained from three captive individuals housed in Kumakumaen (Akita, Japan). The animals included two males and one female. One male (approximately 23 years old, estimated 130 kg) was sampled during hibernation in January 2025, and the other male (8 years old, 118 kg) was sampled during the active season in July 2025. The female (approximately 22 years old) was sampled twice: once during hibernation in January 2025 (estimated 95 kg), and again during the active season in July 2025 (78.3 kg). The bears were maintained under husbandry conditions described in previous studies [26, 27]. Under deep anesthesia, the quadriceps femoris muscle was surgically excised and immediately transported to the laboratory in Dulbecco's Modified Eagle Medium (DMEM) on ice.

### 2.2 Satellite Cell Isolation and Culture

SCs were isolated according to a method described by Yoshioka et al. [28] Briefly, the skeletal muscles (hindlimb muscles including lower leg, thigh, and part of gluteal muscles in mice, chipmunks, and hamsters; lower leg muscles in rats; and a part of quadriceps femoris in bears) were dissected, minced with sterile surgical scissors, and digested in 0.2% type II collagenase solution at 37°C for 60 min. The tissue was gently homogenized using a 20‐G syringe needle and filtered through a 40 μm nylon mesh strainer. The filtrate was centrifuged at 500 × g for 5 min, and the pellet was resuspended in growth medium (GM) consisting of DMEM supplemented with 30% fetal bovine serum, 1% chick embryo extract, 10 ng/mL basic fibroblast growth factor, and 1× penicillin–streptomycin solution. SCs were purified using a classical preplating method, followed by sequential replating to remove fibroblasts and enrich SCs. Purified SCs were plated on Matrigel‐coated culture dishes and maintained at 37°C in GM in a standard 5% CO_2_ incubator. The medium was changed every other day. To induce differentiation, GM was replaced with a differentiation medium (DM) consisting of DMEM supplemented with 2% horse serum and 1× penicillin–streptomycin. Differentiation was induced after 3 days of culture in GM, and the cells were maintained in DM for up to 4 days, depending on the experiment.

### 2.3 Cold Exposure and Rewarming

To induce cold stress, SCs were precultured at 37°C for 48 h, followed by 24 h in GM supplemented with 100 mM HEPES (pH 7.4) (GM‐HEPES) at 37°C. HEPES was added to stabilize the pH during cold exposure and prevent temperature‐dependent pH fluctuations. Plastic containers with small amounts of water were preequilibrated in a conventional refrigerator maintained at 4°C and monitored using a thermometer. The SC culture dishes were placed in these containers and stored at 4°C for 24–48 h without medium change. For rewarming, the SCs were returned to 37°C for 24 h prior to the analysis.

### 2.4 Cell Death and Cytotoxicity Assays

SCs were incubated with PI (5 μg/mL in PBS) for 5 min at room temperature under dark conditions, fixed with 4% paraformaldehyde (PFA) in PBS for 10 min, and counterstained with 4′,6‐diamidino‐2‐phenylindole (DAPI). Fluorescence images were obtained using a BZ‐X800 microscope system. LDH activity in the culture medium was measured using an LDH Cytotoxicity Assay Kit according to the manufacturer's instructions. A blank control consisting of medium without cells was used to determine the background absorbance. Absorbance was measured at 490 nm using a Multiskan GO microplate reader.

### 2.5 Cell Imaging of Reactive Oxygen Species, Intracellular Iron, and Lipid Peroxide

Cold‐induced oxidative stress was assessed using a ROS Assay Kit (Highly Sensitive DCFH‐DA). FerroOrange was used to detect intracellular ferrous iron (Fe^2+^) as a marker of ferroptosis. Lipid peroxide was visualized using Liperfluo. Cells were stained with working concentrations of DCFH‐DA, 1 μM FerroOrange, or 1 μM Liperfluo according to the manufacturer's instructions, and imaged using a BZ‐X800 fluorescence microscope system.

### 2.6 Inhibitor Assays

To analyze the cell death pathways during cold exposure, the following inhibitors were added: 1 μM Ferrostatin‐1 (ferroptosis), 20 μM Necrostatin‐1 (necroptosis), and 20 μM Z‐VAD‐FMK (apoptosis). DMSO was used as the vehicle control. Mouse SCs were cultured at 37°C for 48 h, followed by a 24 h pre‐incubation in GM‐HEPES at 37°C. Cells were then subjected to cold exposure at 4°C for 24 h in the presence of vehicle or inhibitors. Inhibitor‐containing GM‐HEPES was used throughout the 24 h cold treatment period.

### 2.7 Western Blot Analysis

The cell samples were lysed in a 1× concentration of sample buffer and collected using a cell scraper. The lysates were triturated using a 29G syringe needle and heated at 95°C for 5 min. Equal volumes of the lysates were separated using a precast polyacrylamide gel system and transferred to PVDF membranes. The membranes were blocked and incubated with appropriate dilutions of primary and secondary antibodies for detecting GPX4 and myosin heavy chain (MyHC). ACTB and GAPDH served as loading controls to confirm the equivalent loading. Detection was performed using chemiluminescence reagents and images were acquired and quantified using a C‐DiGit Blot Scanner with Image Studio Digits 5.2 software.

### 2.8 EdU Incorporation Assay

Cell proliferation was measured using the Click‐iT EdU Cell Proliferation Kit for Imaging and Alexa Fluor 488 dye following the manufacturer's protocol. EdU (10 μM) was added during the final 6 h of each experimental condition (control, cold exposure at 4°C, or rewarming at 37°C) while maintaining the respective environmental settings. Cells were fixed in 4% PFA for 15 min at room temperature and permeabilized with 0.5% Triton X‐100 in PBS for 20 min. The Click‐iT reaction cocktail was then added and incubated in the dark at room temperature for 30 min. The cells were counterstained with Hoechst 33342 for 30 min in the dark and washed with PBS. Fluorescence images were acquired using a BZ‐X800 microscope system, and the cells were counted using the Hybrid Cell Count module of the BZ‐X Analyzer software based on Hoechst and Alexa Fluor 488 signals in five randomly selected nonoverlapping fields.

### 2.9 RNA Sequencing and Bioinformatics

Total RNA was extracted using ISOGEN II and the RNeasy Mini Kit. Total RNA quantitation and quality checks for purity and integrity were performed using a 5300 Fragment Analyzer System. RNA libraries and transcriptome sequencing were performed by BGI (Guangdong, China). Briefly, libraries were prepared using the Optimal Dual‐mode mRNA Library Prep Kit according to the manufacturer's recommended protocol. Each library was sequenced using the DNBSEQ‐G400 platform with a paired‐end sequence length of 150 bp (PE 150). DEGs were filtered based on a > 2.0‐fold increase or < 1/2 decrease (|log2FC| ≥ 1) and *Q* value < 0.05. Bioinformatics analysis, including GO analysis and heat mapping, was performed using the Dr. Tom software provided by BGI (Guangdong, China). The RNA‐seq datasets generated during the current study were deposited in the Gene Expression Omnibus under accession code GSE296910.

### 2.10 Immunofluorescence Staining of the Cultured Cells

The cells were fixed with 4% PFA, permeabilized with 0.3% Triton X‐100, and blocked with 5% goat serum. The cell samples were then incubated overnight at 4°C with primary antibodies diluted to the appropriate concentrations. Secondary antibodies conjugated with Alexa Fluor 488 or 594 were used appropriately, considering the combination of the host animal species and immunoglobulin subtypes of the primary antibodies. The cells were then treated with a DAPI solution for nuclear counterstaining. All images were obtained using a BZ‐X800 microscope system (Keyence, Osaka, Japan).

### 2.11 Differentiation Index and Myotube Diameter Measurements

Five nonoverlapping areas were randomly selected and used for the image analysis of differentiation index, myotube diameter, and myotube area. The differentiation index was calculated according to the formula [(# nuclei in MyHC‐positive cells/# total DAPI‐positive nuclei)*100]. The myotube diameter was evaluated using the method described by Trendelenburg et al. [29] Briefly, 20 to 30 biggest myotubes were measured in each area. Then, the 10 biggest myotubes from each picture were selected, and the average value (i.e., the mean of 10 myotubes per picture, for a total of 50 biggest myotubes) was considered one biological replicate. The MyHC‐positive regions were defined as myotube area.

### 2.12 In Vivo Muscle Regeneration

Regeneration of skeletal muscle was induced by intramuscular injection of CTX, a snake venom‐derived toxin that causes acute myofiber damage. During the periodic arousal phase of hibernation, 100 μL of 10 μM CTX in PBS was injected into the tibialis anterior (TA) muscle under isoflurane inhalation anesthesia. As a control, the contralateral TA muscle was injected with an equal volume of sterile saline. Skeletal muscle tissue was harvested 7 days postinjection during deep torpor. Animals were first euthanized via intraperitoneal administration of medetomidine (0.6 mg/kg), midazolam (4 mg/kg), and butorphanol (5 mg/kg). After confirmation of deep anesthesia, the animals were decapitated using a rodent guillotine, and tissues were promptly collected.

### 2.13 Histology and Immunostaining of Muscle Tissue

TA muscle samples were frozen in isopentane cooled with liquid nitrogen. They were sectioned into 10‐μm‐thick sections using a cryostat (Microm NX50H or Cryostat HM525NX, Thermo Fisher Scientific, Rockford, IL, USA) and stored at −80°C until further analysis. The sections were fixed in 4% PFA and stained with Mayer's hematoxylin solution for 5 min, followed by eosin staining for 5 min. To identify granulocytes in skeletal muscle, histochemical staining with Naphthol AS‐D chloroacetate esterase (CAE) was performed. CAE staining was carried out using a commercially available kit in accordance with the manufacturer's instructions. After the completion of the esterase reaction, sections were counterstained with Safranin O for 2 min to highlight nuclei, cytoplasmic and extracellular matrix components. For immunohistochemistry, sections were fixed with 4% PFA, permeabilized with 0.1% Triton X‐100, and blocked with 1% bovine serum albumin. The samples were incubated overnight at 4°C with primary antibodies diluted to appropriate concentrations. Secondary antibodies conjugated with fluorescence labeling were used appropriately, considering the combination of the host animal species and immunoglobulin subtypes of the primary antibodies. The antibodies and their combinations are listed in Table S2. To detect M1‐like and M2‐like macrophages, a fluorescent dye‐conjugated CD68 antibody and an antibody labeling kit for rabbit IgG (FlexAble 2.0 CoraLite Plus 555 Antibody Labeling Kit for Rabbit IgG) were used to accommodate the shared host species. Sections were mounted with DAPI Fluoromount‐G for nuclear counterstaining. Images were obtained using a BZ‐X800 microscope system (Keyence, Osaka, Japan). Five to six nonoverlapping areas were randomly selected and uniformly distributed across each muscle section for the quantitative analysis.

### 2.14 Quantification and Statistical Analysis

Quantitative analyses included cell viability, EdU incorporation, immunofluorescence signal intensity, muscle fiber cross‐sectional area, and macrophage infiltration. At least five randomly selected, nonoverlapping fields per sample were analyzed using the BZ‐X Analyzer software with the Hybrid Cell Count module (Keyence). The average value per sample was considered one biological replicate. For comparisons between two groups, an unpaired two‐tailed *t*‐test with Welch's correction was performed. One‐way or two‐way analysis of variance (ANOVA) followed by Tukey's multiple comparison test was used to compare more than two groups or two independent variables. Because some experiments involved small sample sizes, formal normality tests (e.g., Shapiro–Wilk) were interpreted with caution. Assumptions for ANOVA were primarily evaluated through residual diagnostics (QQ plots and residual–fitted plots). For robustness, pairwise contrasts identified as significant by ANOVA/Tukey were additionally examined with Welch's *t*‐tests, which yielded the same qualitative interpretation of group differences. All statistical analyses and graph generation were performed using GraphPad Prism (version 10, GraphPad Software). All data are presented as mean ± standard deviation. The significance thresholds were defined as *p* < 0.05, *p* < 0.01, *p* < 0.001, and *p* < 0.0001, respectively. The detailed statistical methods, *n* values, and group comparisons are described in the corresponding figure legends.

## 3 Results

### 3.1 Isolation and Characterization of Satellite Cells From Hibernating Mammals

To the best of our knowledge, SCs had not previously been isolated from mammalian hibernators before this study. Here, we established primary SC cultures from skeletal muscle of mammalian hibernators following the method of Yoshioka et al. [28] The isolated SCs showed high purity, as evidenced by the presence of Pax7‐ or MyoD‐positive cells, which accounted for over 95% of the primary cells isolated from the hamster skeletal muscle (Figure S1A–C). SCs efficiently differentiated into myotubes when cultured in DM, which confirmed their myogenic potential (Figure S1D,E). Using the same method, we also isolated SCs from skeletal muscle of chipmunks and bears (Figure S1F,G).

### 3.2 Cold Resistance and Proliferative Capacity of Satellite Cells From Hibernating Hamsters

**FIGURE 1:**
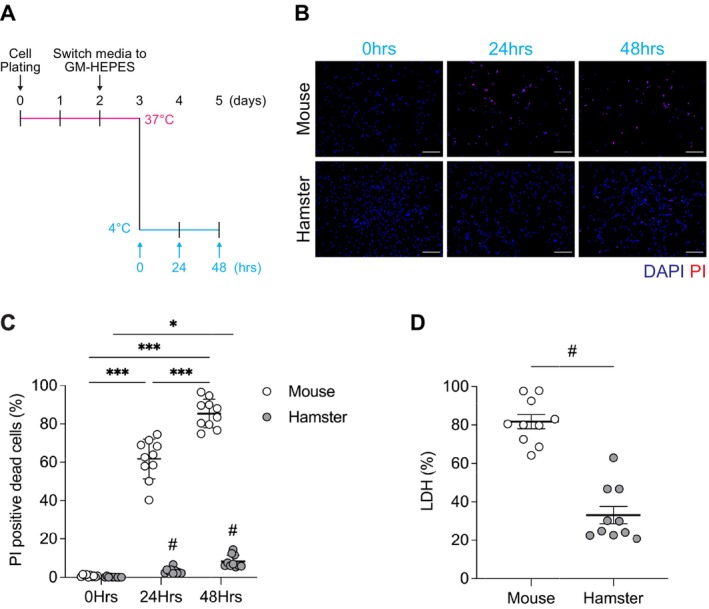
Satellite cells from hibernating hamsters exhibit resistance to cold‐induced cell death. (A) Schematic overview of the experimental design to assess cold‐induced cell death (CICD) in skeletal muscle satellite cells (SCs). SCs isolated from mice and Syrian hamsters were cultured at 37°C for 48 h after plating, followed by a medium change to a growth medium supplemented with HEPES (GM‐HEPES) and an additional 24 h incubation at 37°C. The cells were then subjected to cold exposure at 4°C for 24 or 48 h, without changing the medium. (B) Representative fluorescence images of SCs stained with propidium iodide (PI, red) and 4′,6‐diamidino‐2‐phenylindole (DAPI, blue) following cold exposure. PI‐positive nuclei are frequently observed in mouse SCs, whereas hamster SCs largely retain their viability. (C, D) Quantification of cell death by PI staining (C) and lactate dehydrogenase (LDH) release (D). In mouse SCs, both the PI‐positive cell counts and LDH release increased significantly over time. Hamster SCs showed minimal changes. Data are presented as mean ± SD (*n* = 10 per group). Statistical significance was assessed using two‐way ANOVA, followed by Tukey's post hoc test. *p < 0.05, **p < 0.01, ***p < 0.001 for time‐dependent comparisons within species. #*p* < 0.05, for comparisons between mouse and hamster at the same time point. Scale bars: 200 μm (B).

We determined whether SCs from mammalian hibernators exhibit resistance to CICD. In SCs from mice, cold treatment for 24 and 48 h resulted in 61.7% and 85.4% propidium iodide (PI)‐positive dead cells, respectively (Figure 1A–C). Similarly, marked CICD was observed in SCs from nonhibernating rats (Figure S2A–C). In contrast, SCs from the Syrian hamster, a small hibernating species, exhibited pronounced resistance to CICD, with only 3.2% and 8.2% of PI‐positive cells after 24 and 48 h of cold exposure, respectively (Figure 1B,C). Consistently, in the LDH cytotoxicity assay, hamster SCs exhibited marked resistance, whereas mouse SCs showed over 80% cell death after 24 h of cold exposure (Figure 1D). We have also observed CICD resistance in SCs isolated from other hibernators (Figure S2A–C), including chipmunks, smaller hibernators that undergo deep torpor and drastic *T*_b_ oscillations [30], and bears, a larger hibernator that experiences sustained reductions in T_b_ during hibernation [6]. These results suggest that CICD resistance is a conserved cellular feature in SCs of mammalian hibernators that undergo torpor‐associated reduction in *T*_b_.

**FIGURE 2:**
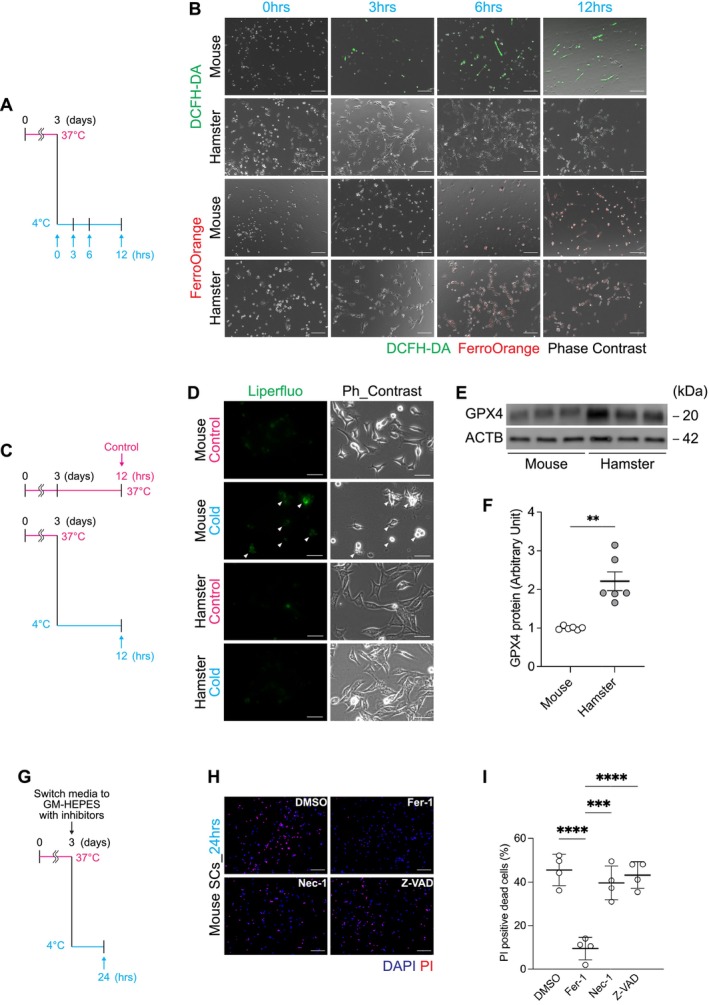
Cold‐induced cell death in mouse satellite cells is mediated by ferroptosis and suppressed in hamster cells. (A) Schematic overview of the experimental design used to evaluate oxidative stress and ferroptosis in satellite cells (SCs) exposed to cold conditions. Mouse and hamster SCs were incubated at 4°C for 3, 6, or 12 h, followed by staining with DCFH‐DA and FerroOrange to detect reactive oxygen species (ROS) and intracellular free iron levels, respectively. (B) Representative fluorescence images of DCFH‐DA and FerroOrange staining of mouse and hamster SCs after 0–12 h of cold exposure. (C) Mouse and hamster SCs were incubated at 4°C for 12 h, followed by staining with Liperfluo to detect intracellular lipid peroxides. (D) Representative fluorescence (Liperfluo) and phase‐contrast images of mouse and hamster SCs after cold exposure. White arrowheads indicate detaching cells that show enhanced Liperfluo fluorescence, reflecting increased lipid peroxide accumulation. (E, F) Western blot analysis (E) and quantification (F) of glutathione peroxidase 4 (GPX4) and β‐actin (ACTB) in mouse and hamster SCs under baseline conditions. *n* = 6 per group. Protein levels were expressed as arbitrary units. (G) Schematic overview of the experimental design for the inhibitor assays. Mouse SCs were cultured at 37°C for 48 h, followed by a 24 h pre‐incubation in GM‐HEPES at 37°C. Cells were then subjected to cold exposure at 4°C for 24 h in the presence of vehicle (DMSO), ferroptosis inhibitor (Ferrostatin‐1), necroptosis inhibitor (Necrostatin‐1), or apoptosis inhibitor (Z‐VAD‐FMK). (H) Representative fluorescence images of PI and DAPI staining in the presence of each inhibitor. (I) Quantification of PI‐positive cells in each group. Data are presented as mean ± SD (*n* = 4 per group). Statistical significance was assessed using one‐way ANOVA followed by Tukey's post hoc test. *p < 0.05, **p < 0.01, ***p < 0.001, ****p < 0.0001 vs. DMSO‐treated control. Scale bars: 200 μm (B and H), 50 μm (D).

To determine the role of ferroptosis in CICD, we assessed markers of cellular stress following cold exposure in mouse and hamster SCs. Hallmarks of ferroptosis, including increased reactive oxygen species (ROS) production (DCFH‐DA staining) and accumulation of intracellular free iron (FerroOrange), were observed in mouse SCs after 3–6 h of cold exposure, whereas these responses were minimal in hamster SCs (Figure 2A,B). Lipid peroxidation associated with CICD (assessed by Liperfluo) was also apparent in mouse SCs, most notably in detached dead cells, while the cold treatment had minimal effect on hamster SCs (Figure 2C,D). The level of GPX4 protein, an antioxidant enzyme that inhibits ferroptosis, was more abundant in hamster SCs compared with that in mouse SCs (Figure 2E,F). Neither necroptosis inhibitor Necrostatin‐1 nor apoptosis inhibitor Z‐VAD‐FMK prevented CICD in mouse SCs, whereas the ferroptosis‐specific inhibitor Ferrostatin‐1 rescued cell viability (Figure 2G–I). These results indicated that CICD in nonhibernating mouse SCs is mediated by ferroptosis, whereas resistance in hamster SCs is attributed to the suppression of ferroptosis, which may be mediated by intrinsically higher GPX4 expression.

**FIGURE 3:**
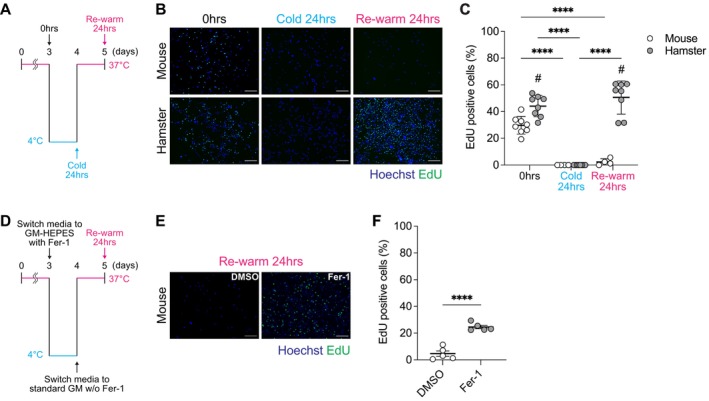
Satellite cells from hibernating hamsters resume proliferation after cold exposure and rewarming. (A) Schematic overview of the experimental design to assess satellite cell (SC) proliferative capacity after cold exposure and rewarming. SCs isolated from mice and Syrian hamsters were cultured at 37°C for 48 h after plating, and then incubated in GM‐HEPES at 37°C for 24 h, followed by cold exposure at 4°C for 24 h. The cells were subsequently rewarmed and maintained at 37°C for 24 h. 5‐ethynyl‐2′‐deoxyuridine (EdU) was added during the final 6 h to label proliferating cells. (B) Representative fluorescence images of Hoechst and EdU staining of mouse and hamster SCs under control, cold exposure, and rewarming conditions. (C) Quantification of EdU‐positive cells under the indicated conditions. Data are presented as mean ± SD (*n* = 4–8 per group). (D) Mouse SCs were cultured at 37°C for 48 h, followed by a 24 h pre‐incubation in GM‐HEPES at 37°C. Cells were then subjected to cold exposure at 4°C for 24 h in the presence of vehicle (DMSO) or ferroptosis inhibitor (Ferrostatin‐1). The cells were subsequently rewarmed and maintained at 37°C for 24 h in standard GM without DMSO or Ferrostatin‐1. EdU was added during the final 6 h. (E) Representative fluorescence images of Hoechst and EdU staining of mouse SCs after rewarming. (F) Quantification of EdU‐positive cells in each treatment. Data are presented as mean ± SD (*n* = 5 per group). Statistical significance was assessed using two‐way ANOVA, followed by Tukey's post hoc test. ****p < 0.0001 for comparisons between time points. # *p* < 0.05, for comparisons between mouse and hamster at the same time point. Scale bars: 200 μm (B and E).

To further examine the cellular response to cold stress, we pulsed cells with the thymidine analog 5‐ethynyl‐2′‐deoxyuridine (EdU) to assess cell proliferation. After 24 h of cold exposure, EdU‐positive proliferating cells were absent in mouse and hamster SCs. Upon rewarming, mouse SCs exhibited minimal proliferation, whereas hamster SCs restored proliferation to levels comparable to those of preexposure conditions (Figure 3A–C). These results indicate that hamster SCs resist CICD while retaining their capacity to proliferate following cold exposure and rewarming. Importantly, mouse SCs treated with Fer‐1 during cold exposure showed comparable EdU incorporation upon rewarming to that of the pre‐cold control group (Figure 3D–F). This demonstrates that mouse SCs are able to maintain their proliferative capacity during cold exposure through CICD inhibition by suppressing ferroptosis.

### 3.3 RNA‐Seq Revealed Cold‐Induced Downregulation of Myogenic Genes in Hamster SCs

**FIGURE 4:**
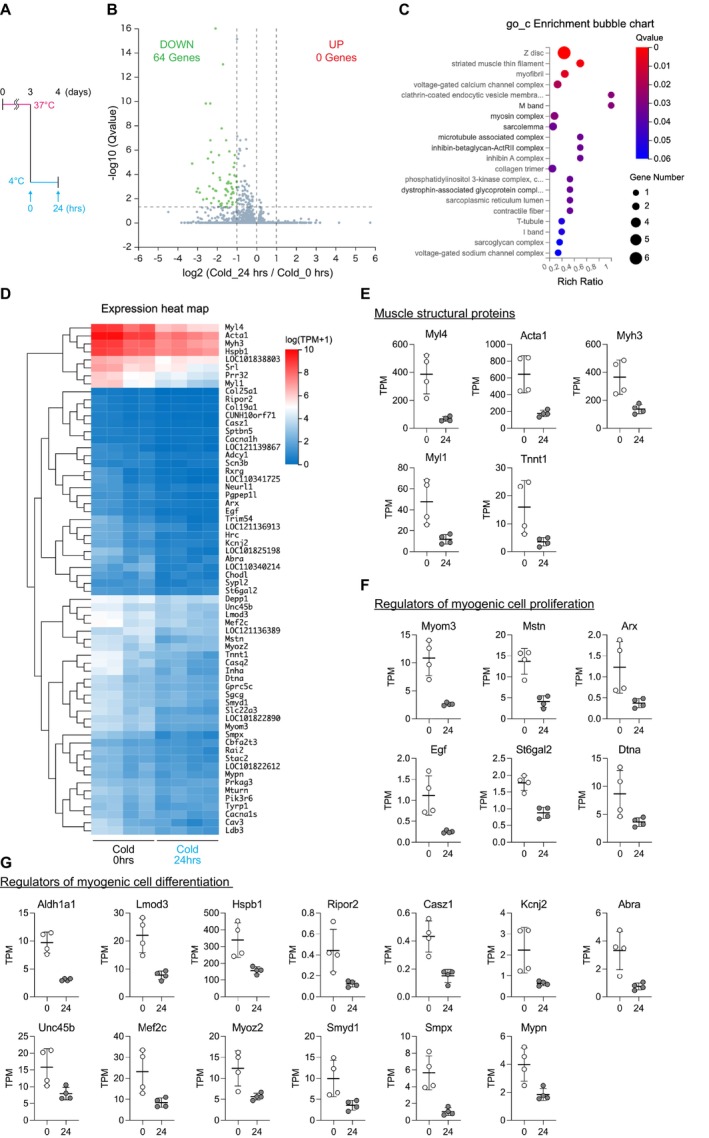
Cold exposure induces broad downregulation of myogenesis‐related genes in hamster satellite cells. (A) Schematic overview of the experimental design used for transcriptome analysis. Satellite cells (SCs) isolated from Syrian hamsters were cultured at 37°C for 48 h after plating, followed by 24 h incubation in GM‐HEPES at 37°C, and exposure to 4°C for 24 h. Total RNA was extracted for RNA sequencing before and after exposure to cold conditions. (B) Volcano plot showing differentially expressed genes (DEGs) between control and cold‐exposed SCs. A total of 64 genes were significantly downregulated after cold exposure (|log2 fold change| ≥ 1, *Q* < 0.05); no genes were upregulated. (C) Gene Ontology (GO) enrichment analysis (cellular component category) of 64 downregulated DEGs. (D) Heatmap displaying the expression levels of all 64 downregulated DEGs in the control and cold‐exposed SCs. (E–G) Selected subsets of downregulated DEGs were manually curated based on prior literature and known gene functions. (E) Genes encoding the structural components of muscle fibers. (F) Genes involved in the regulation of satellite cell proliferation. (G) Genes associated with myogenic differentiation. RNA‐seq was performed with biological replicates (*n* = 4 per group). Differential expression was defined as |log2 fold change| ≥ 1 and *Q* < 0.05. For panels (E–G), gene expression levels are shown as transcripts per million (TPM) values directly derived from RNA‐seq output without further normalization.

RNA‐seq analysis was performed to analyze the cellular responses of hamster SCs to 24 h of cold exposure, and 64 differentially expressed genes (DEGs) were identified. DEGs were defined as those with a fold change of |log2 FC| ≥ 1 (i.e., more than 2‐fold upregulation or less than half expression) and a *Q*‐value < 0.05. All 64 DEGs were downregulated in response to cold exposure (Figure 4A,B). Gene Ontology (GO) enrichment analysis (cellular component category) revealed significant enrichment for terms associated with striated muscle structures, including “Z‐disc,” “striated muscle thin filament,” “myofibril,” “M band,” “myosin complex,” and “sarcolemma” (Figure 4C). The 64 DEGs included genes encoding the structural components of muscle fibers (Myl4, Acta1, Myh3, Myl1, and Tnnt1), regulators of myogenic cell proliferation [Myom3 [31], Mstn [32], Arx [33], Egf [34], St6gal2 [35], and Dtna [36]], and genes associated with myogenic differentiation [Aldh1a1 [37], Lmod3 [38], Hspb1 [39, 40], Ripor2 [41], Casz1 [42], Kcnj2 [43], Abra [44], Unc45b [45], Mef2c [46], Myoz2 [47], Smyd1 [48], Smpx [49], and Mypn [50]] (Figure 4D–G). These results indicated that cold exposure induces broad transcriptional suppression of genes associated with muscle structure, SC activation, and myogenic differentiation in hamster SCs. This suggests a molecular mechanism that involves downregulation of SC function under cold conditions.

### 3.4 Cold Exposure Suppresses Myogenic Activation and Differentiation in Hamster SCs

**FIGURE 5:**
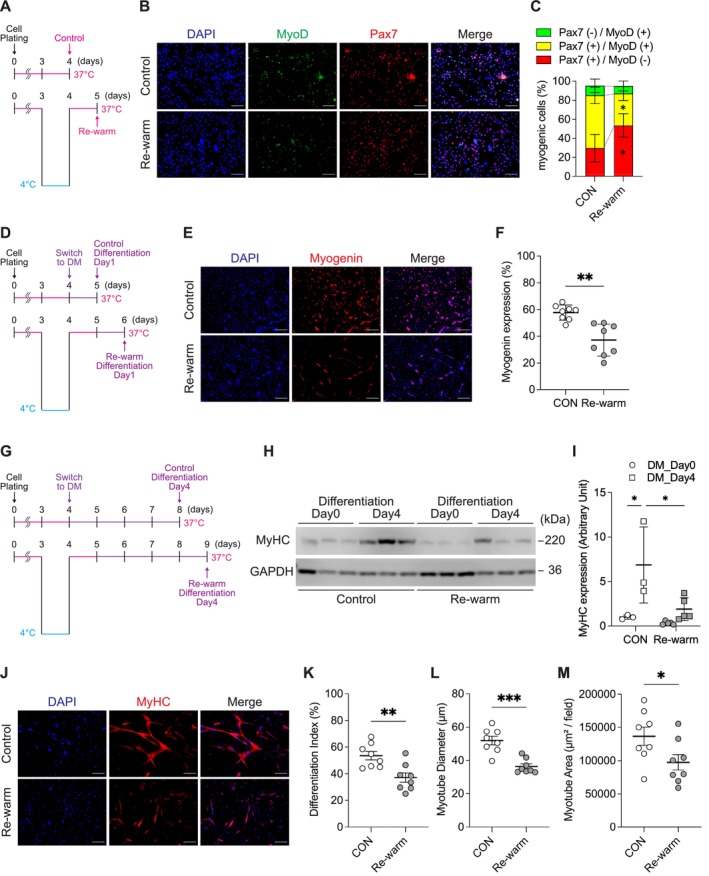
Cold exposure suppresses satellite cell activation and differentiation in hamster cells. (A) Schematic overview of experimental design for cold exposure and rewarming to evaluate satellite cell activation. Hamster satellite cells (SCs) were cultured at 37°C for 48 h after plating and then incubated in GM‐HEPES at 37°C for 24 h, followed by 24 h exposure to 4°C. The cells were then rewarmed and maintained at 37°C for 24 h before analysis. (B) Representative fluorescence images of Pax7 and MyoD immunostaining in the control and rewarmed SCs. (C) Quantification of the relative proportions of three cell populations—Pax7^+^/MyoD^−^, Pax7^+^/MyoD^+^, and Pax7^−^/MyoD^+^—within the total cell population. (D) Schematic overview of the experimental design for cold exposure, rewarming, and the subsequent induction of differentiation. (E) Representative images of myogenin immunostaining in control and rewarmed SCs after 24 h in the differentiation medium. (F) Quantification of myogenin‐positive cells after 24 h of differentiation. (G) Schematic overview of the experimental design for cold exposure, rewarming, and the subsequent induction of differentiation. During the differentiation period, cells were maintained in DM for 4 days, with the medium replaced with fresh DM every other day. (H) Western blot analysis of myosin heavy chain (MyHC) and glyceraldehyde‐3‐phosphate dehydrogenase (GAPDH) protein levels on differentiation days 0 and 4. GAPDH was used as a loading control. (I) Quantification of the MyHC protein band intensity. (J) Representative images of MyHC immunostaining. (K) Quantification of differentiation index. (L) Quantification of the myotube diameter. (M) Quantification of the total area of MyHC‐positive regions. Statistical analyses were performed as follows: (F), (K), (L), and (M), unpaired two‐tailed Welch's *t*‐test; (I) two‐way ANOVA followed by Tukey's post hoc test. Sample sizes: (F), (K), and (L), *n* = 8 per group; (I), *n* = 3–5 per group. Data are presented as mean ± SD. *p < 0.05, **p < 0.01, ***p < 0.001; significance levels correspond to comparisons indicated in each panel. #*p* < 0.05, for comparisons between the control and rewarmed groups at the same time point. Scale bars: 200 μm (B, E, and J).

To determine the effect of cold exposure on myogenic progression in CICD‐resistant hamster SCs, we measured the expression of Pax7 and MyoD in cells rewarmed after 24 h of cold treatment. Compared with the noncold‐exposed controls, the proportion of MyoD‐positive cells, a marker of SC activation, was significantly reduced in cold‐exposed hamster SCs, whereas the proportion of Pax7‐positive/MyoD‐negative (Pax7^+^/MyoD^−^) quiescent satellite cells was concomitantly increased (Figure 5A–C). In addition, the percentage of myogenin‐positive cells, a marker of myogenic differentiation, significantly decreased following cold exposure in hamster SCs subjected to differentiation induction (Figure 5D–F). Furthermore, despite being subjected to the same duration of differentiation induction, typical hallmarks of late‐stage differentiation including the protein expression of the myosin heavy chain, differentiation index, myotube diameter, and myotube area were all reduced, indicating inhibition of the myogenic process in response to cold exposure (Figure 5G–M).

### 3.5 Hibernation Delays Skeletal Muscle Regeneration

**FIGURE 6:**
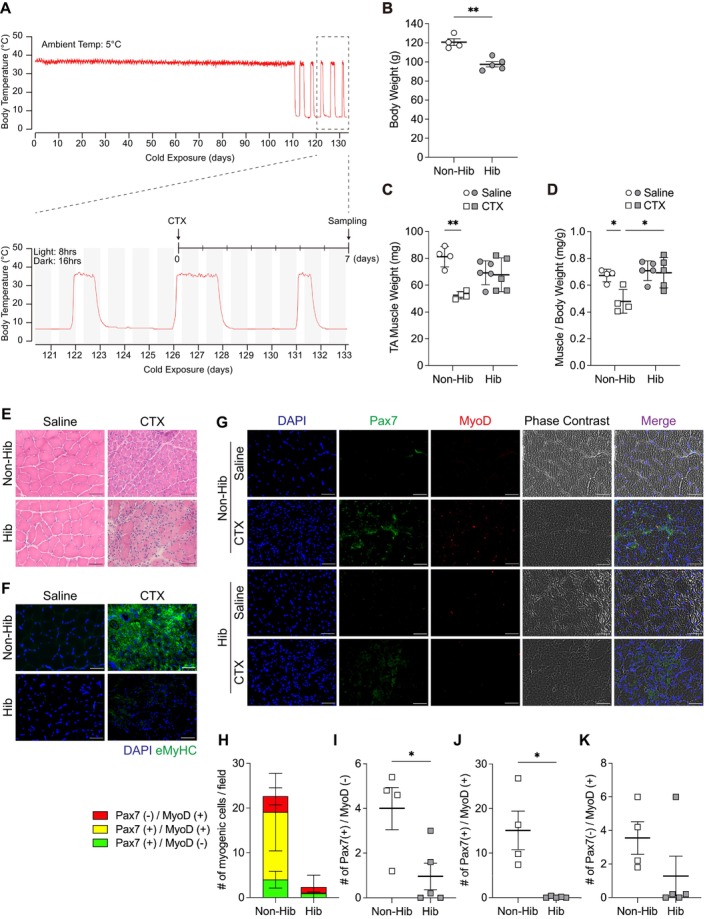
Skeletal muscle regeneration is suppressed during hibernation in Syrian hamsters. (A) Representative body temperature fluctuations recorded in a Syrian hamster during hibernation. The period corresponding to hibernation was enlarged to indicate the timing of cardiotoxin (CTX) injection during interbout arousal and tissue collection 7 days later during deep torpor. (B) Body weights of nonhibernating (Non‐Hib) and hibernating (Hib) animals at the time of tissue collection. (C) Absolute weights of the tibialis anterior (TA) muscles, with saline injected into one leg and CTX injected on the contralateral side. (D) TA muscle weight normalized to body weight. (E) Representative hematoxylin and eosin staining of TA cross‐sections. (F) Representative immunofluorescence images of embryonic myosin heavy chain expression. (G) Representative images showing Pax7 (green) and MyoD (red) expression along with DAPI staining and phase contrast; merged images are shown. (H) Total number of Pax7^+^ and/or MyoD^+^ cells per field. For each sample, at least five random fields were imaged, and the average number of positively stained cells per field was used as one biological replicate. (I–K) Separate quantification of Pax7^+^/MyoD^−^ (I), Pax7^+^/MyoD^+^ (J), and Pax7^−^/MyoD^+^ (K) cell populations in Non‐Hib and Hib groups. Statistical analysis: (B, I–K), unpaired two‐tailed Welch's *t*‐test; (C, D) two‐way ANOVA followed by Tukey's post hoc test. Sample sizes: Non‐Hib, *n* = 4; Hib, *n* = 5. Data are presented as mean ± SD. *p < 0.05, **p < 0.01. Scale bars: 50 μm (E–G).

Having established that cold exposure of primary cultured SCs from Syrian hamsters suppressed myogenic progression, we determined the effect of hibernation on skeletal muscle regeneration in vivo. Syrian hamsters were transferred from a standard environment (ambient temperature: 23°C ± 1°C, light 14 h, dark 10 h) to a winter‐like environment (ambient temperature: 5°C, light 8 h, dark 16 h), where they entered hibernation over a period of 2–4 months. During hibernation, the animals repeatedly cycled between states of deep torpor, marked by a pronounced reduction in *T*_b_, and periodic arousal, during which *T*_b_ returned to normothermic levels (Figure 6A).

Compared with nonhibernating controls housed in the same cold and short‐day conditions, hibernating hamsters exhibited reduced body weight (Figure 6B). Following 7 days of cardiotoxin (CTX) injection into the TA muscle, nonhibernating controls showed a significant reduction in muscle weight compared to the contralateral saline‐injected side, whereas muscle weight did not decrease in the hamsters during hibernation (Figure 6C,D). Histological analysis on Day 7 postinjury revealed numerous regenerating myofibers with small diameters and central nuclei in the nonhibernating group, whereas the hibernating group exhibited limited fiber regeneration, with persistent cellular presence in the interstitial space, which suggested delayed or arrested regenerative progression (Figure 6E). The expression of embryonic MyHC (eMyHC), a marker of regenerating fibers, was observed 7 days after injury in the nonhibernating group but was absent in hamsters during hibernation (Figure 6F). Furthermore, the proportion of Pax7‐ or MyoD‐positive SCs significantly increased during regeneration in nonhibernating controls; however, this enhancement was markedly suppressed during hibernation (Figure 6G–K). These in vivo findings demonstrate that hibernation, characterized by cycles of deep torpor and arousal, suppresses regenerative activation of SCs and delays muscle regeneration following injury.

### 3.6 Delayed and Attenuated Inflammatory Response During Muscle Regeneration in Hibernating Animals

**FIGURE 7:**
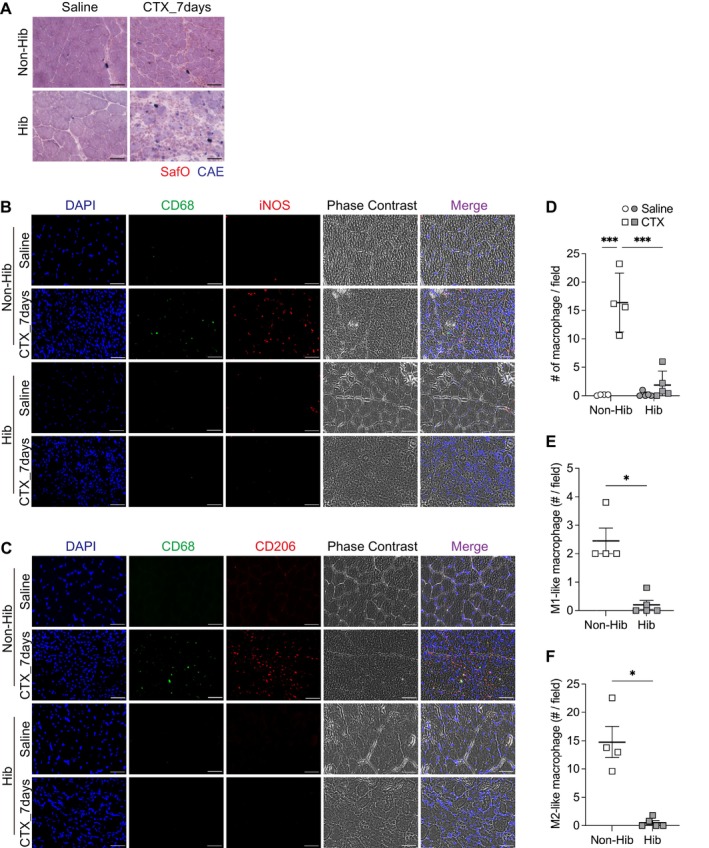
Macrophage infiltration is suppressed during hibernation in regenerating muscle. (A) Representative histochemical images of tibialis anterior muscle sections stained with Naphthol AS‐D chloroacetate esterase (CAE; blue) to label granulocytes, with Safranin O counterstaining (red) to highlight cytoplasmic and extracellular matrix components. In CTX‐treated muscles at Day 7, marked infiltration of CAE‐positive granulocytes (blue) is observed, particularly in the hibernating (Hib) group. (B, C) Representative immunofluorescence images stained for CD68 (pan‐macrophage marker), iNOS (M1 marker; panel B), CD206 (M2 marker; panel C), DAPI, and phase contrast. (D) Quantification of total macrophages, defined as CD68^+^/DAPI^+^ cells, in both saline‐ and CTX‐injected muscles. (E) Quantification of M1‐like macrophages, defined as CD68^+^/iNOS^+^/DAPI^+^ cells. (F) Quantification of M2‐like macrophages, defined as CD68^+^/CD206^+^/DAPI^+^ cells. For quantification, at least five randomly selected fields were imaged per sample. The number of marker‐positive cells per field was averaged and used as one biological replicate. Statistical analysis: (D) two‐way ANOVA followed by Tukey's post hoc test; (E, F) unpaired two‐tailed Welch's *t*‐test, performed only on CTX‐injected samples because of undetectable marker expression in saline‐injected muscles. Sample sizes: Non‐Hib, *n* = 4; Hib, *n* = 5. Data are presented as mean ± SD. *p < 0.05, ***p < 0.001. Scale bar: 50 μm (A–C).

Regulation of the tissue microenvironment, particularly immune cell infiltration, plays an important role in muscle regeneration [51]. In nonhibernating controls, granulocyte infiltration, including neutrophils, was evident at an early stage after CTX‐induced injury (e.g., Day 3) and had largely resolved by Day 7 (Figure S3). In contrast, granulocytes (cholinesterase‐positive round‐shaped cells) remained detectable within the injured region at Day 7 in the hibernating group, indicating a delayed resolution of acute inflammation during hibernation (Figure 7A). In addition, in nonhibernating controls, the number of CD68‐positive macrophages was significantly increased in the damaged muscle at 7 days following CTX injection, whereas macrophage infiltration was markedly reduced during hibernation. Both proinflammatory M1‐like macrophages (CD68‐positive/iNOS‐positive) and anti‐inflammatory M2‐like macrophages (CD68‐positive/CD206‐positive) were sparsely detected in the injured muscles of the hibernating group (Figure 7B–F). These results suggest that hibernation suppresses both SC activation and myogenic regeneration while simultaneously delaying and attenuating the inflammatory response, which is essential for effective muscle repair.

## 4 Discussion

In this study, we isolated SCs from the skeletal muscles of mammalian hibernators and examined their response to cold exposure. Our main findings were as follows: (1) SCs isolated from hibernating species, including Syrian hamsters, chipmunks, and bears, exhibited resistance to CICD; (2) their proliferation was completely arrested under cold conditions but resumed upon rewarming; (3) cold exposure downregulated the expression of genes associated with myogenic activation and differentiation; and (4) cold‐induced suppression of myogenic processes was observed in vivo during hibernation following experimentally induced muscle injury.

In the present study, these observations were mechanistically linked to a marked resistance to CICD by suppressing ferroptosis, an iron‐dependent form of regulated cell death. This intrinsic cellular process likely represents a mechanism that enables hibernating animals to survive periods of low *T*_b_ associated with hibernation at cold temperatures. In contrast, cold exposure significantly suppressed SC activation and differentiation in vitro, and delayed muscle regeneration in vivo. Moreover, immune cell infiltration into damaged muscle tissue was markedly delayed and reduced during hibernation. These results suggest that during hibernation, a state characterized by a profound reduction in *T*_b_, multiple acute cellular responses, including myogenesis and inflammation, were concurrently downregulated.

Previous studies have shown that culturing muscle SCs or myoblasts at subphysiological temperatures (e.g., 30°C–37°C) delays proliferation and differentiation, potentially leading to the formation of smaller myofibers [52, 53, 54]. Although our findings are generally consistent with temperature‐dependent suppression of myogenesis, the culture conditions used in the present study (exposure to 4°C) represent a far more extreme cold challenge. Under these conditions, SC proliferation was completely arrested, but proliferative capacity was restored upon rewarming. This reversible suppression highlights a unique aspect of SC behavior in hibernating animals, in which cold exposure temporarily blocks proliferation without inducing irreversible cell cycle arrest. Interestingly, this occurred despite the marked suppression of canonical myogenic regulators such as MyoD. This suggests that SC proliferation can occur independently of MyoD expression, which is consistent with previous reports indicating that MyoD‐negative SCs are capable of division under certain conditions such as mechanical overload, in which sustained HeyL expression suppresses MyoD while promoting SC expansion [55].

Mechanistically, resistance to CICD in SCs from hibernators may be attributed, in part, to the intrinsically higher expression of GPX4, a key regulator of ferroptosis. GPX4 is an antioxidant enzyme that prevents lipid peroxidation, and its elevated expression in SCs from hibernating animals likely contributes to the suppression of ferroptotic cell death during cold exposure. This mechanism may represent an important aspect of cellular resilience that allows SCs to remain viable under extreme cold stress conditions [17, 18]. In support of this idea, our results demonstrated that pharmacological inhibition of ferroptosis with Ferrostatin‐1 preserved the proliferative capacity of cold‐exposed mouse SCs upon rewarming. While mouse SCs normally exhibit minimal proliferation after cold exposure, Ferrostatin‐1 treatment allowed them to recover proliferation to levels comparable to pre‐cold conditions. These data indicate that suppression of CICD is sufficient to maintain SC functional integrity during transient cold stress even in nonhibernating mammals. Although additional work will be required, these findings raise the possibility that regulating ferroptosis susceptibility under cold conditions could improve hypothermic preservation strategies for cells and tissues of nonhibernating mammals.

We also observed a concomitant cold‐induced increase in the proportion of Pax7^+^/MyoD^−^ SCs, suggesting preservation of the quiescent SC pool. Maintaining quiescence is important for long‐term muscle homeostasis and regenerative capacity [56]. Therefore, the suppression of SC activation during cold exposure may represent a protective strategy that safeguards the stem cell reserve during prolonged environmental stress, thus enabling future regeneration once favorable conditions return.

Our in vivo experiments demonstrated that muscle regeneration was delayed during hibernation, which is consistent with previous studies on 13‐lined ground squirrels [57]. In that model, delayed muscle regeneration during hibernation was associated with the reduced expression of transforming growth factor beta 1, a key profibrotic factor in skeletal muscle, along with minimal fibrosis. This suggests that a slower regenerative response may contribute to the preservation of muscle integrity, while avoiding pathological remodeling. Our results extend these observations by demonstrating that suppression of muscle regeneration in hibernating animals is accompanied by delayed granulocyte infiltration and markedly reduced macrophage recruitment following injury. This persistent granulocyte presence may explain the increased interstitial cellularity observed at Day 7 despite reduced macrophage abundance. Macrophages play an important role in promoting muscle regeneration through metabolic and signaling interactions with SCs [58]. Thus, the attenuation of regenerative and inflammatory responses during hibernation likely reflects physiological adaptation to reduce the metabolic expenditure associated with tissue remodeling. The physiological significance of these adaptations likely resides in the minimization of energy expenditure during periods when energy is limited. During hibernation, the suppression of metabolically demanding processes may represent a physiological adaptation that conserves energy and supports the maintenance of tissue integrity under cold and nutrient‐poor conditions.

Although our RNA‐seq analysis revealed downregulation of myogenesis‐related genes following cold exposure, the specific molecular pathways mediating these transcriptional changes remain to be identified. Future studies examining the epigenetic mechanisms, chromatin remodeling, and transcriptional regulation underlying cold‐induced gene suppression will be important to determine how cold exposure alters the myogenic program in SCs, and may ultimately provide insight into the adaptive regulation of SC function in hibernating animals. Understanding these pathways may contribute to the development of novel approaches for muscle regeneration or preservation under conditions of reduced activity or metabolic suppression such as aging, disuse, or therapeutic hypothermia.

### 4.1 Limitation

Although this study examined the impact of cold on skeletal muscle stem cell viability and cellular behavior, it is important to acknowledge that hibernation involves additional physiological stressors. These include hypoxia, nutrient deprivation, and reduced tissue perfusion, all of which may influence stem cell function and muscle remodeling in vivo. Further studies are necessary to clarify how these stressors influence the regulation of regenerative processes during hibernation.

## Author Contributions

M.M. conceived and designed the project; T.M., R.K., M.M., Y.T., M.S., T.T., D.T., G.L., S.K., Y.W., and M.W. acquired, analyzed, and interpreted the data; T.M., R.K., Y.Y., M.W., and M.M. wrote, revised, and edited the paper. M.M. directed the research project.

## Funding

This work was supported by MEXT KAKENHI Grant Numbers 25K22761, 24H02013, 23K18432, 23K24697 to M.M., 23H04940 to Y.Y., and M.W., 23H04939 to Y.Y., and was supported by The Nakatomi Foundation, The Takeda Science Foundation, and The Hiroshima University Fund “Nozomi H Foundation” subsidy for the promotion of cancer treatment research. All funding agencies approved the broad elements of study design during the process of grant submission and review and played no direct role in the design of the study, collection, analysis, and interpretation of data, and in writing the manuscript.

## Conflicts of Interest

The authors declare no conflicts of interest.

## Supporting information


**Data S1:** fsb271297‐sup‐0001‐DatasetS1.xlsx.

**Figure S1:** Characterization of satellite cells isolated from hibernating animals.

**Figure S2:** Cold‐induced cell death in satellite cells from additional hibernating and nonhibernating species.

**Figure S3:** Histochemical analysis of immune cell infiltration during muscle regeneration following CTX injury.

**Figure S4:** Original western blotting images & Detailed experimental conditions.

**Table S1:** List of materials, reagents, and equipment.

**Table S2:** Antibodies and labeling kits used for immunofluorescence and immunohistochemistry.

## Data Availability

The data that support the findings of this study are available in the Materials and Methods, Results, and/or Supporting Information of this article.
