# Supplementary material for: Cold‐Induced Suppression of Myogenesis in Skeletal Muscle Stem Cells Contributes to Delayed Muscle Regeneration During Hibernation
Source: FASEB J. 2025 Dec 1;39(23):e71297. doi: 10.1096/fj.202502651R (PMC12668025; doi:10.1096/fj.202502651R)
Supplement: Supplementary file 2 — Figure S1: Characterization of satellite cells isolated from hibernating animals. [file FSB2-39-e71297-s004.pdf]

Supplemental Figure 1

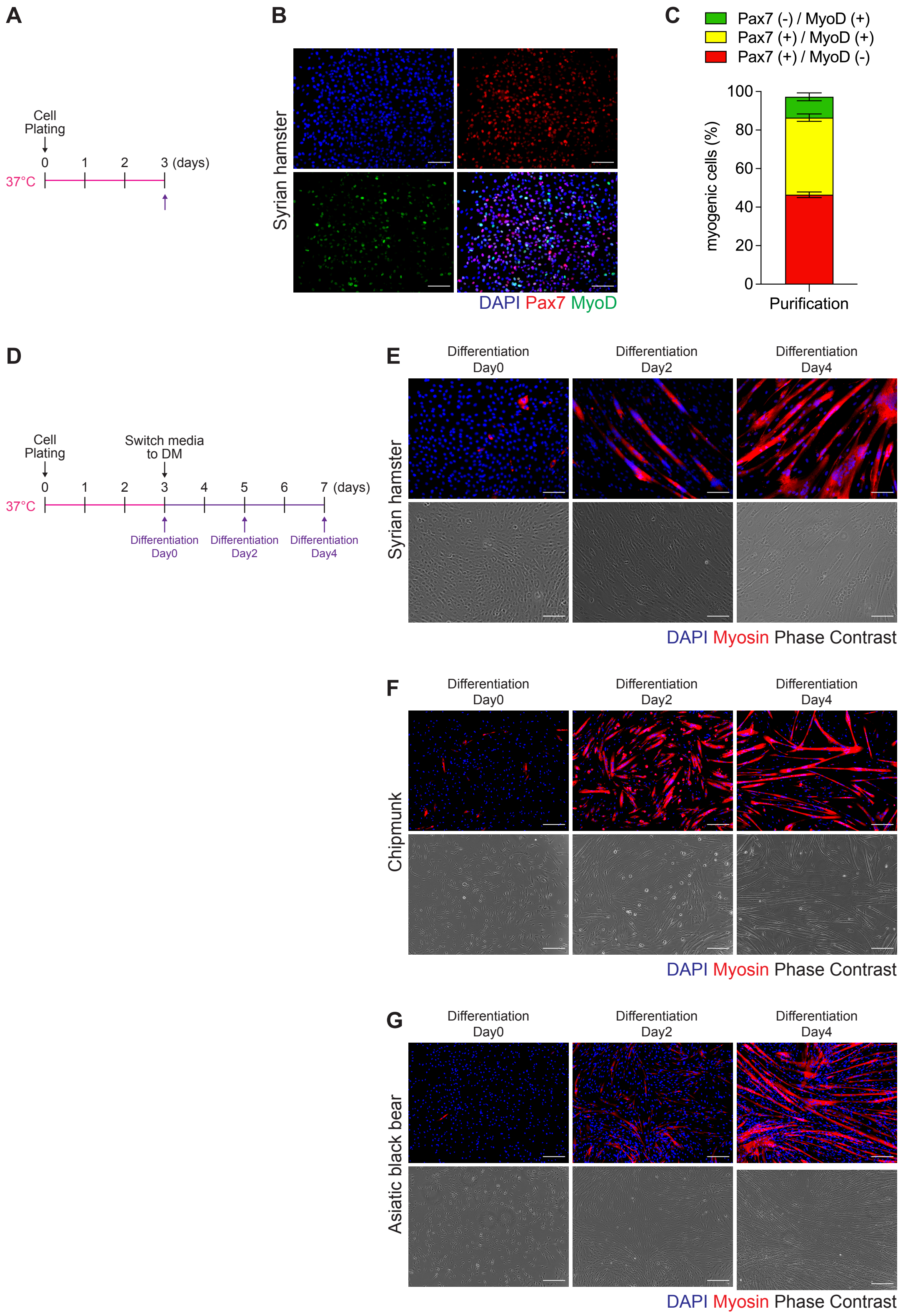

Supplemental Figure 1 Characterization of satellite cells isolated from hibernating animals

(A) Schematic overview of experimental design for satellite cell (SC) isolation and characterization. SCs were plated immediately after isolation and maintained for three days at 37 °C, with the growth medium replaced every other day. (B) Representative immunofluorescence images showing DAPI (blue), Pax7 (red), and MyoD (green) staining of the Syrian hamster SCs. (C) Quantification of the $Pax7^{+}/MyoD^{-}$, $Pax7^{+}/MyoD^{+}$, and $Pax7^{-}/MyoD^{+}$ cell populations. Each value represents the proportion relative to total cell number. Pax7- and/or MyoD-positive cells accounted for over 95% of the total, indicating high purity of the isolated SC population (n = 6). (D) Schematic overview of the experimental design for SC differentiation. The growth medium was switched to differentiation medium (DM) on day 3 post-plating and DM was replaced every other day. (E) Representative images of Syrian hamster SCs induced to differentiate, showing DAPI (blue), myosin heavy chain (red), and phase contrast. (F, G) Representative images of chipmunk SCs (F) and bear SCs (G) after differentiation under the same conditions, indicating that their myogenic potential was preserved. Scale bar: 200 μm (B, E, F, and G).
