# Supplementary material for: Cold‐Induced Suppression of Myogenesis in Skeletal Muscle Stem Cells Contributes to Delayed Muscle Regeneration During Hibernation
Source: FASEB J. 2025 Dec 1;39(23):e71297. doi: 10.1096/fj.202502651R (PMC12668025; doi:10.1096/fj.202502651R)
Supplement: Supplementary file 3 — Figure S2: Cold‐induced cell death in satellite cells from additional hibernating and nonhibernating species. [file FSB2-39-e71297-s002.pdf]

Supplemental Figure 2

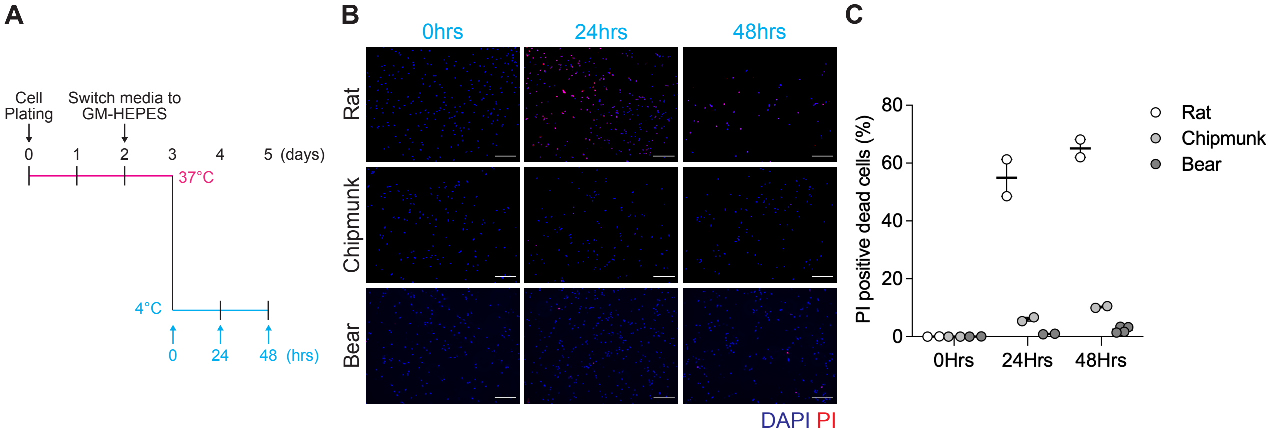

Supplemental Figure 2 Cold-induced cell death in satellite cells from additional hibernating and nonhibernating species

(A) Schematic overview of the experimental design used to assess cold-induced cell death (CICD) in skeletal muscle satellite cells (SCs) from rats (nonhibernators) and chipmunks (hibernators). SCs were cultured at 37 °C for 48 h after plating, followed by a medium change to GM-HEPES, and an additional 24 h incubation at 37 °C. Cells were then subjected to cold exposure at 4 °C for 24 or 48 h without changing the medium. (B) Representative fluorescence images showing DAPI (blue) and propidium iodide (PI; red) staining at each time point. (C) Quantification of PI-positive cells at different time points. Rat SCs (n = 2 per time point) exhibited a time-dependent increase in PI-positive nuclei, whereas SCs from chipmunks (n = 2 per time point) and bears (n = 2 at 0 h and 24 h, n = 4 at 48 h) showed minimal change. Data are presented as mean ± SD from 2–4 biological replicates, depending on species and time point. Statistical analyses were not performed owing to the limited sample size. Scale bar: 200 µm (B).
