# Supplementary material for: Cold‐Induced Suppression of Myogenesis in Skeletal Muscle Stem Cells Contributes to Delayed Muscle Regeneration During Hibernation
Source: FASEB J. 2025 Dec 1;39(23):e71297. doi: 10.1096/fj.202502651R (PMC12668025; doi:10.1096/fj.202502651R)
Supplement: Supplementary file 4 — Figure S3: Histochemical analysis of immune cell infiltration during muscle regeneration following CTX injury. [file FSB2-39-e71297-s001.pdf]

Supplemental Figure 3

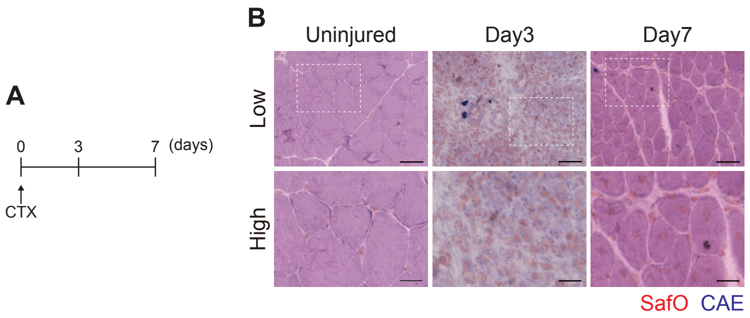

Supplemental Figure 3 Histochemical analysis of immune cell infiltration during muscle regeneration following CTX injury

(A) Schematic overview of the experimental design. Tibialis anterior (TA) muscles were injected with cardiotoxin (CTX) and collected at the indicated time points (day 0, day 3, and day 7). (B) Representative histochemical images of TA muscle sections stained with Naphthol AS-D chloroacetate esterase (CAE; blue) to label granulocytes, with Safranin O counterstaining (red) to highlight cytoplasmic and extracellular matrix components. Images are shown at low and high magnification, with the high-magnification panels corresponding to the regions outlined by white dashed rectangles in the low-magnification images. Granulocyte infiltration is apparent at day 3 and largely resolves by day 7. Scale bar: 50 μm (low magnification), 20 μm (high magnification).
