# Supplementary material for: Cold‐Induced Suppression of Myogenesis in Skeletal Muscle Stem Cells Contributes to Delayed Muscle Regeneration During Hibernation
Source: FASEB J. 2025 Dec 1;39(23):e71297. doi: 10.1096/fj.202502651R (PMC12668025; doi:10.1096/fj.202502651R)
Supplement: Supplementary file 6 — Table S1: List of materials, reagents, and equipment. [file FSB2-39-e71297-s003.pdf]

Category	Product/Kit Name	Manufacturer	Catalog No.	Application
Animal Care	Isoflurane Inhalation Solution	VIATRIS	871119	Anesthesia
Animal Care	Isoflurane	FUJIFILM Wako Pure Chemical Corporatio	099-06571	Anesthesia
Animal Care	Pentobarbital Sodium Salt	NACALAI TESQUE	26427-72	Anesthesia
Animal Care	Medetomidine Hydrochloride (Dorbene vet)	Kyoritsu Seiyaku Corp	QG03AA01	Anesthesia
Animal Care	Midazolam (Midazolam Injection 10mg[SANDOZ])	Sandoz K.K.	N05CD08	Anesthesia
Animal Care	Butorphanol tartrate (Vetorphale)	Meiji Animal Health	N02AF01	Anesthesia
Animal Care	Rodents Diets	Oriental Yeast	MF	Diets
Animal Care	Nosan Corporation Laboratory Animal Feed	Nihon Nosan	Labo MR Standa	Diets
Animal Care	Cardiotoxin	LATOXAN	L8102	Muscle regeneration
Animal Care	iButton	Analog Devices	DS1922L-F5#	Temperature logger
Antibodies	Anti-Pax7	Santa Cruz Biotechnology	sc-81648	Immunofluorescence
Antibodies	Anti-MyoD	Santa Cruz Biotechnology	sc-760	Immunofluorescence
Antibodies	Myog Antibody (F5D)	Developmental Studies Hybridoma Bank	F5D	Immunofluorescence
Antibodies	MYH1E Antibody (MF 20)	Developmental Studies Hybridoma Bank	MF 20	Immunofluorescence
Antibodies	MYH3 Antibody (F1.652)	Developmental Studies Hybridoma Bank	F1.652	Immunofluorescence
Antibodies	CoraLite Plus 488-conjugated CD68 Polyclonal antibody	Proteintech	CL488-28058	Immunohistochemistry
Antibodies	CD206 Polyclonal antibody	Proteintech	18704-1-AP	Immunohistochemistry
Antibodies	Anti-iNOS	FUJIFILM Wako Pure Chemical Corporatio	015-28371	Immunohistochemistry
Antibodies	Goat anti-Mouse IgG (H+L) Cross-Adsorbed Secondary Antibody, Alexa Fluor 594	Thermo Fisher Scientific	A-11005	Secondary antibody
Antibodies	Goat anti-Rabbit IgG (H+L) Cross-Adsorbed Secondary Antibody, Alexa Fluor 488	Thermo Fisher Scientific	A-11008	Secondary antibody
Antibodies	Goat anti-Mouse IgG1 Cross-Adsorbed Secondary Antibody, Alexa Fluor 488	Thermo Fisher Scientific	A-21121	Secondary antibody
Antibodies	Goat anti-Mouse IgG2b Cross-Adsorbed Secondary Antibody, Alexa Fluor 594	Thermo Fisher Scientific	A-21145	Secondary antibody
Antibodies	CoraLite594 - conjugated Goat Anti-Rabbit IgG(H+L)	Proteintech	SA00013-4	Secondary antibody
Antibodies	Anti-Glutathione Peroxidase 4 antibody [EPNCIR144]	abcam	ab125066	Western blot
Antibodies	Beta Actin Monoclonal antibody	Proteintech	66009-1-Ig	Western blot
Antibodies	GAPDH (6C5)	Santa Cruz Biotechnology	sc-32233	Western blot
Antibodies	Peroxidase AffiniPure Goat Anti-Rabbit IgG (H+L)	Jackson ImmunoResearch	111-035-003	Western blot
Antibodies	Peroxidase AffiniPure Goat Anti-Mouse IgG (H+L)	Jackson ImmunoResearch	115-035-003	Western blot
Instruments	Multiskan GO Microplate Reader	Thermo Fisher Scientific	1510	Absorbance measurement
Instruments	BZ-X800 Microscope System	Keyence	BZ-X800	Imaging
Instruments	5300 Fragment Analyzer System	Agilent Technologies	5300	RNA quality check
Instruments	DNBSEQ-G400 Sequencer	MGITech	DNBSEQ-G400	RNA-seq
Instruments	Cryostat	Thermo Fisher Scientific	NX50	Tissue sectioning
Instruments	Cryostat	Thermo Fisher Scientific	HM525NX	Tissue sectioning
Instruments	C-DiGit Blot Scanner	LI-COR Biosciences	3600-00	Western blot imaging
Reagents/Kits	FlexAble CoraLite Plus 488 Antibody Labeling Kit for Rabbit IgG	Proteintech	KFA001	Antibody labeling
Reagents/Kits	FlexAble 2.0 CoraLite Plus 555 Antibody Labeling Kit for Rabbit IgG	Proteintech	KFA502	Antibody labeling
Reagents/Kits	Z-VAD-FMK	Selleck	S7023	Apoptosis inhibitor
Reagents/Kits	Penicillin-Streptomycin Solution (×100)	FUJIFILM Wako Pure Chemical Corporatio	168-23191	Cell culture
Reagents/Kits	D-MEM(High Glucose) with L-Glutamine, Phenol Red and Sodium Pyruvate	FUJIFILM Wako Pure Chemical Corporatio	043-30085	Cell culture
Reagents/Kits	-Cellstain- PI solution	Dojindo	P378	Cell death assay
Reagents/Kits	-Cellstain- DAPI solution	Dojindo	D523	Cell death assay
Reagents/Kits	4%-Paraformaldehyde Phosphate Buffer Solution	NACALAI TESQUE	09154-56	Cell death assay
Reagents/Kits	Cytotoxicity LDH Assay Kit-WST	Dojindo	343-91753	Cytotoxicity assay
Reagents/Kits	FerroOrange	Dojindo	342-09533	Ferroptosis detection
Reagents/Kits	Ferrostatin-1	Cayman Chemical	17729	Ferroptosis inhibitor
Reagents/Kits	Chick Embryo Extract, Ultrafiltrate, Liquid (CEE)	USBiological	C3999	Growth medium
Reagents/Kits	Recombinant Murine FGF-basic	PeproTech	450-33	Growth medium
Reagents/Kits	Isopentane	NACALAI TESQUE	26404-75	Histology
Reagents/Kits	Mayer's hematoxylin solution	MUTO PURE CHEMICALS	88591	Histology
Reagents/Kits	1% Eosin Y Solution	MUTO PURE CHEMICALS	32002	Histology
Reagents/Kits	Safranin O Stock Solution	MUTO PURE CHEMICALS	41101	Histology
Reagents/Kits	Naphthol AS-D chloroacetate Esterase staining kit	MUTO PURE CHEMICALS	15702	Histology
Reagents/Kits	Triton X-100	NACALAI TESQUE	35501-15	Immunofluorescence
Reagents/Kits	DAPI-Fluoromount-G	SouthernBiotech	0100-20	Immunohistochemistry
Reagents/Kits	Liperfluo	Dojindo	L248	Lipid peroxidation imaging
Reagents/Kits	Necrostatin-1	Cayman Chemical	11658	Necroptosis inhibitor
Reagents/Kits	Click-iT EdU Cell Proliferation Kit for Imaging, Alexa Fluor 488 dye	Thermo Fisher Scientific	C10337	Proliferation assay
Reagents/Kits	ISOGEN II	NIPPON GENE	311-07361	RNA extraction
Reagents/Kits	RNeasy Mini Kit	Qiagen	74104	RNA extraction
Reagents/Kits	ROS Assay Kit -Highly Sensitive DCFH-DA	Dojindo	340-09811	ROS detection
Reagents/Kits	Collagenase Type II	Worthington Biochemical Corporation	CLS2	SC isolation
Reagents/Kits	Matrigel	Corning	354230	SC isolation
Reagents/Kits	Injection Needle Pink 18G	Terumo	NN-1838R	SC isolation
Reagents/Kits	Cell Strainer	AS ONE	VCS-40	SC isolation
Reagents/Kits	Collagen TypeI coated Dish 60mm	AGC TECHNO GLASS	4010-010	SC isolation
Reagents/Kits	Dimethyl Sulfoxide	NACALAI TESQUE	13408-64	Solvent
Reagents/Kits	MyJector Needle Implantable Type Insulin Syringe 29G	Terumo	SS-10M2913	Western blot
Reagents/Kits	Sample Buffer Solution with 3-Mercapto-1,2-propanediol (×4)	FUJIFILM Wako Pure Chemical Corporatio	196-16142	Western blot
Reagents/Kits	Any kD Mini-PROTEAN TGX Precast Protein Gels	Bio-Rad Laboratories	4569036	Western blot
Reagents/Kits	Trans-Blot Turbo RTA Mini 0.2 μm PVDF Transfer Kit	Bio-Rad Laboratories	1704272	Western blot
Reagents/Kits	E-R520L e-PAGEL	ATTO Corporation	2331730	Western blot
Reagents/Kits	ClearTrans SP PVDF Membrane, Hydrophobic, 0.2μm	FUJIFILM Wako Pure Chemical Corporatio	033-22453	Western blot
Reagents/Kits	Bullet Blocking One for Western Blotting	NACALAI TESQUE	13779-01	Western blot
Reagents/Kits	Blocking One	NACALAI TESQUE	03953-95	Western blot
Reagents/Kits	ImmunoStar LD	FUJIFILM Wako Pure Chemical Corporatio	290-69904	Western blot
Reagents/Kits	ImmunoStar Zeta	FUJIFILM Wako Pure Chemical Corporatio	295-72404	Western blot

Supplemental Table 1. List of materials, reagents, and equipment

Detailed list of animal care materials, cell culture reagents, chemicals, staining reagents, antibodies, molecular biology kits, and other laboratory equipment. The table includes the product names, manufacturers, catalog numbers, and specific applications, as referenced in the Materials and Methods section.
