# Supplementary material for: Cold‐Induced Suppression of Myogenesis in Skeletal Muscle Stem Cells Contributes to Delayed Muscle Regeneration During Hibernation
Source: FASEB J. 2025 Dec 1;39(23):e71297. doi: 10.1096/fj.202502651R (PMC12668025; doi:10.1096/fj.202502651R)
Supplement: Supplementary file 7 — Table S2: Antibodies and labeling kits used for immunofluorescence and immunohistochemistry. [file FSB2-39-e71297-s007.pdf]

Sample	Antigen	Primary Antibody	Secondary Antibody	Antibody Labeling Kit	Application
Hamster satellite cells	Pax7	anti-Pax-7, mouse monoclonal, IgG1 kappa light chain	Goat anti-Mouse IgG (H+L), Alexa Fluor 594	–	Immunofluorescence
Hamster satellite cells	MyoD	anti-MyoD, rabbit polyclonal, IgG	Goat anti-Rabbit IgG (H+L), Alexa Fluor 488	–	Immunofluorescence
Hamster satellite cells	Myogenin	anti-Myogenin, mouse monoclonal, IgG1 kappa light chain	Goat anti-Mouse IgG (H+L), Alexa Fluor 594	–	Immunofluorescence
Hamster satellite cells	Myosin heavy chain, sarcomere (MHC)	anti-Myosin heavy chain, mouse monoclonal, IgG2b, kappa light chain	Goat anti-Mouse IgG2b, Alexa Fluor 594	–	Immunofluorescence
Hamster skeletal muscle tissue	Myosin heavy chain (embryonic)	anti-embryonicMHC, mouse monoclonal, IgG1	Goat anti-Mouse IgG1, Alexa Fluor 488	–	Immunohistochemistry
Chipmunk satellite cells	Myosin heavy chain, sarcomere (MHC)	anti-Myosin heavy chain, mouse monoclonal, IgG2b, kappa light chain	Goat anti-Mouse IgG2b, Alexa Fluor 594	–	Immunofluorescence
Hamster skeletal muscle tissue	Pax7	anti-Pax-7, mouse monoclonal, IgG1 kappa light chain	Goat anti-Mouse IgG1, Alexa Fluor 488	–	Immunohistochemistry
Hamster skeletal muscle tissue	MyoD	anti-MyoD, rabbit polyclonal, IgG	Goat anti-Rabbit IgG (H+L), CoraLite594	–	Immunohistochemistry
Hamster skeletal muscle tissue	CD68	CoraLite Plus 488-conjugated CD68 Polyclonal antibody	–	–	Immunohistochemistry
Hamster skeletal muscle tissue	iNOS	anti-iNOS, rabbit polyclonal, IgG	–	FlexAble 2.0 CoraLite Plus 555 Antibody Labeling Kit for Rabbit IgG	Immunohistochemistry
Hamster skeletal muscle tissue	CD206	anti-CD206, rabbit polyclonal, IgG	–	FlexAble 2.0 CoraLite Plus 555 Antibody Labeling Kit for Rabbit IgG	Immunohistochemistry

Supplemental Table 2. Antibodies and labeling kits used for immunofluorescence and immunohistochemistry

List of primary and secondary antibodies used for immunofluorescence and immunohistochemistry, along with host species and corresponding antibody labeling kits. The table includes antigen targets, antibody specifications, and applications, as referenced in the Materials and Methods.
